# Rhabdomyolysis: early prognostication of renal failure and other adverse outcomes

**DOI:** 10.1186/cc14363

**Published:** 2015-03-16

**Authors:** J Simpson, A Taylor, DK Menon, N Sudhan, A Lavinio

**Affiliations:** 1 University of Cambridge, UK; 2 Cambridge University Hospitals, Cambridge, UK

## Introduction

The clinical diagnosis of rhabdomyolysis is confirmed by creatine kinase (CK) levels >1,000 IU/l [1]. A local therapeutic protocol triggers aggressive renoprotective treatment in all patients with CK >2,000 IU/l. To evaluate local practice and refine CK thresholds for the instigation of renoprotective treatment, we studied the correlation between CK time trends and adverse outcomes such as acute kidney injury (AKI), the need for emergency renal replacement therapy (RRT) and mortality. We also evaluated the McMahon Score, a risk prediction model based on demographic, clinical, and laboratory variables available on admission [2].

## Methods

A retrospective observational study of adults with confirmed rhabdomyolysis admitted to the Neurosciences Critical Care Unit between 2002 and 2012. Data collection included APACHE score, daily CK (with PEAK CK defined as the maximum CK recorded throughout ICU stay), creatinine, calcium, phosphate and bicarbonate levels, AKI, RRT, ICU length of stay and mortality.

## Results

A total of 232 patients met the inclusion criteria. Rhabdomyolysis was associated with trauma (76%), medical (15%) and surgical (9%) admission diagnoses. Forty-five (19%) patients developed AKI, with 29 (12.5%) requiring RRT. Mortality was significantly higher in patients who developed AKI (62% vs. 18%, *P *< 0.001). Average CK on admission was 5,009 IU/l (SD 12,403 IU/l). CK values remained greater than 2,000 IU/l for an average of 3.3 days (range 1 to 10 days). Although PEAK CK was greater in patients requiring RRT compared with those that did not (PEAK CK: 32,354 IU/l vs. 7,353 IU/l, *P *= 0.001), receiver operator characteristic curves revealed that a threshold for PEAK CK >5,000 IU/l is only 55% specific and 83% sensitive for the prediction of need for RRT. CK peaks on the day of admission in 46% of patients, on day 2 in 37%, and on day 3 or later in 17% of cases. A McMahon Score >6 calculated on admission is 68% specific and 86% sensitive for RRT.

## Conclusion

Although higher CK levels are associated with adverse outcomes, instigation of renoprotective treatment should not be based solely on CK levels. A McMahon Score >6 on admission allows for a more sensitive, specific and timely identification of patients at risk of renal failure requiring RRT.
